# Effects of remote ischemic preconditioning on early markers of intestinal injury in experimental hemorrhage in rats

**DOI:** 10.1038/s41598-024-63293-4

**Published:** 2024-06-05

**Authors:** Stefan Hof, Hendrik Untiedt, Anne Hübner, Carsten Marcus, Anne Kuebart, Anna Herminghaus, Christian Vollmer, Inge Bauer, Olaf Picker, Richard Truse

**Affiliations:** Department of Anesthesiology, University Hospital Düsseldorf, Düsseldorf, Germany

**Keywords:** Cardiovascular biology, Circulation, Diagnostic markers, Cardiovascular biology, Cardiovascular diseases, Gastrointestinal diseases, Trauma

## Abstract

The maintenance of intestinal integrity and barrier function under conditions of restricted oxygen availability is crucial to avoid bacterial translocation and local inflammation. Both lead to secondary diseases after hemorrhagic shock and might increase morbidity and mortality after surviving the initial event. Monitoring of the intestinal integrity especially in the early course of critical illness remains challenging. Since microcirculation and mitochondrial respiration are main components of the terminal stretch of tissue oxygenation, the evaluation of microcirculatory and mitochondrial variables could identify tissues at risk during hypoxic challenges, indicate an increase of intestinal injury, and improve our understanding of regional pathophysiology during acute hemorrhage. Furthermore, improving intestinal microcirculation or mitochondrial respiration, e.g. by remote ischemic preconditioning (RIPC) that was reported to exert a sufficient tissue protection in various tissues and was linked to mediators with vasoactive properties could maintain intestinal integrity. In this study, postcapillary oxygen saturation (µHbO_2_), microvascular flow index (MFI) and plasmatic d-lactate concentration revealed to be early markers of intestinal injury in a rodent model of experimental hemorrhagic shock. Mitochondrial function was not impaired in this experimental model of acute hemorrhage. Remote ischemic preconditioning (RIPC) failed to improve intestinal microcirculation and intestinal damage during hemorrhagic shock.

## Introduction

Despite the development of therapeutic concepts like permissive hypotension, the early anticipation of trauma induced coagulopathy and the implementation and establishment of standardized trauma algorithms, death by hemorrhage represents a global burden with about 2 million deaths per year worldwide^1^. Especially the first days after trauma seem to be critical. Gunst et al. estimated 50% of injury-associated deaths to occur within the first 30 days after trauma^2^. In the 1980s Trunkey et al. described the distribution of early death after a severe injury as a trimodal concept^3^. According to this model, death occurs immediately after the initial event, in the early course after administration to a local trauma center, or after several days in the intensive care units. Whereas immediate and early deaths are caused by the consequence of direct trauma, cardiovascular failure and neurological impairment^4^, the main cause for later deaths is the development of inflammation, sepsis and multi-organ failure^5^. In contrast to immediate and early deaths, active management of the late phase turned out to have the largest potential to reduce total mortality in trauma patients^4^.

Because of its adrenergic receptor activation, the gastrointestinal tract is one of the most critically perfused vascular beds during acute hemorrhage. Crucial to maintaining the mean arterial perfusion pressure of vital organs, intestinal vasoconstriction and hypoperfusion may impair tissue integrity, enable the translocation of bacterial agents into the systemic circulation and induce local inflammation. Both, bacterial translocation and regional inflammation, are reported to induce sepsis and multi organ failure^6^. In intensive care units intestinal dysfunction occurs frequently^7^. Among critically ill patients mortality increases markedly, when gastrointestinal function is compromised^8^. Therefore, the implementation of gastrointestinal tissue protection and maintenance of intestinal barrier function represent important goals to avoid secondary complications in the later course of traumatic injury and hemorrhagic shock. Since microcirculation and mitochondrial respiration represent the terminal stretch of regional tissue oxygenation, microvascular and mitochondrial measurements could serve as early, dynamic and possibly more accurate markers of intestinal tissue injury. Unfortunately, studies investigating microcirculation and mitochondrial respiration only exist for experimental animal models of abdominal sepsis^9,10^. Concordant trials with a simultaneous evaluation of microcirculatory and mitochondrial variables to investigate the local pathophysiology of hemorrhagic shock are missing.

Murry et al. were the first to describe an endogenous protective mechanism that might improve regional microcirculation as well as mitochondrial pathways called ischemic preconditioning (IPC)^11^. In detail, short and non-lethal cycles of ischemia and reperfusion were able to protect the conditioned tissue from a following period of critical index ischemia. IPC is reported to reduce apoptosis in intestinal epithelial cells by enhancing the expression of antiapoptotic proteins and reducing oxidative stress in Wistar rats^12^. Further, IPC promotes physiological cell function and protects against mucosal disorders and gut malfunction^13^. Since enterocytes are an important determinant of the intestinal barrier function and bacterial translocation was indicated to be a cornerstone in the development of multi-organ failure after hypoxic challenges^6^, Aksöyek et al. could show a diminished intestinal tissue damage by IPC after superior mesenteric artery clamping^14^. In this study, intestinal tissue protection by IPC was associated with a lower rate of bacterial translocation into the systemic circulation and organs with high immunological activity like mesenteric lymph nodes, spleen and liver^14^. Nevertheless, IPC is limited in its transferability to the clinical setting due to the invasive manner of pretreatment.

This limitation of IPC was overcome by Przyklenk et al. who found that not only the conditioned but also myocardial tissue that depends on another vascular unit benefit from ischemic preconditioning, a phenomenon called remote ischemic preconditioning (RIPC)^15^. Later, it was reported that the conditioning effect can also be described between different organ systems^16^. In experimental studies RIPC attenuates intestinal mucosal damage after ischemia–reperfusion injury similar to the effect of IPC^17,18^. Further not only remote ischemic preconditioning, but also remote ischemic postconditioning reduced intestinal injury and cytokine release in a model of ischemia–reperfusion^19^, making this therapeutic approach suitable for prevention and therapy of intestinal damage, even if the effectiveness of RIPC might differ depending on the time of application. Many of the mediators suggested to provide the effect of RIPC are produced within the microcirculation, transported as humoral factors or known to have vasoactive properties at a local level of the cardiovascular system^20^. Recently, we reported, that intervening local microvascular balance might be suitable to improve microvascular oxygen supply^21^. Therefore, an improvement of microvascular indices and tissue oxygenation could be the terminal stretch of RIPC-mediated tissue protection and maintain intestinal barrier function in critically ill patients.

Therefore, this study was designed to address the following questions:

(i) Which changes in postcapillary oxygen saturation, perfusion and vessel architecture can be described in a model of fixed-pressure hemorrhagic shock in rats?
(ii) Is mitochondrial respiration impaired in the early course of hemorrhagic shock?
(iii) Do histological and plasmatic markers of intestinal impairment mirror gastrointestinal tissue injury during hemorrhagic shock?
(iv) Does remote ischemic preconditioning exert beneficial effects on microcirculatory variables, mitochondrial respiration and markers of intestinal tissue integrity?

## Results

### Macrocirculation

**Figure 1:**
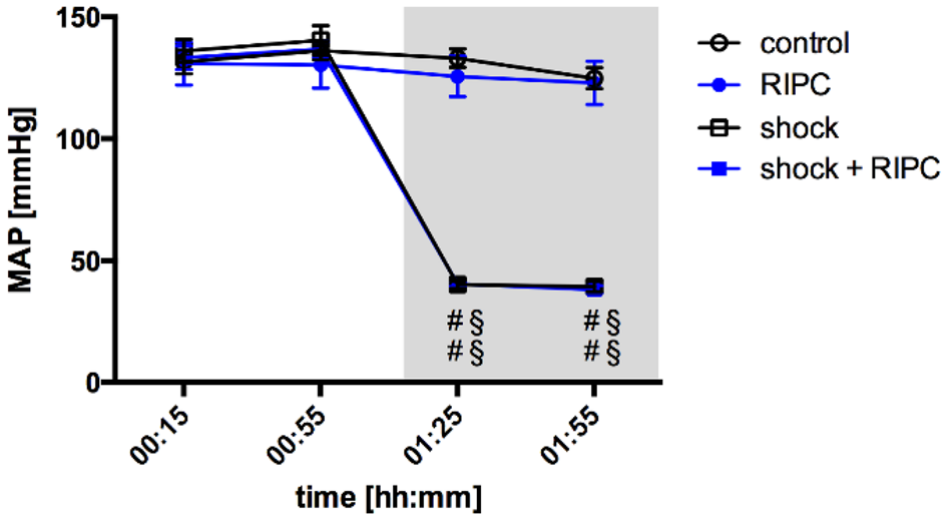
Mean arterial blood pressure. Time-related changes of the mean arterial blood pressure (MAP) in mmHg under physiological and hemorrhagic conditions. Interventional groups received remote ischemic preconditioning treatment (RIPC) and/or fixed-pressure hemorrhage (shock). One hour of acute hemorrhage is marked grey. Data are presented as mean ± SEM for n = 12 Wistar-rats. 2-way ANOVA for repeated measurements followed by Bonferroni post-hoc test did not reveal significant differences between experimental groups with or without RIPC treatment under the same hemodynamic conditions. ^#^p ≤ 0.05 vs. baseline values, ^§^p ≤ 0.05 vs. normovolemic control group with the same RIPC-treatment.

According to the experimental protocol mean arterial pressure (MAP) decreased significantly from 136 ± 5 mmHg to 40 ± 2 mmHg in the early course of hemorrhagic shock. Similarly, in animals receiving RIPC procedure (shock + RIPC) MAP decreased from 133 ± 3 mmHg to 40 ± 1 mmHg. Neither initial blood pressure levels nor values measured during acute hemorrhage differed between shock groups. Animals under normovolemia did not show any significant alteration of blood pressure during the experiments and remained within their initial blood pressure range (Fig. 1).

### Microcirculation

**Figure 2:**
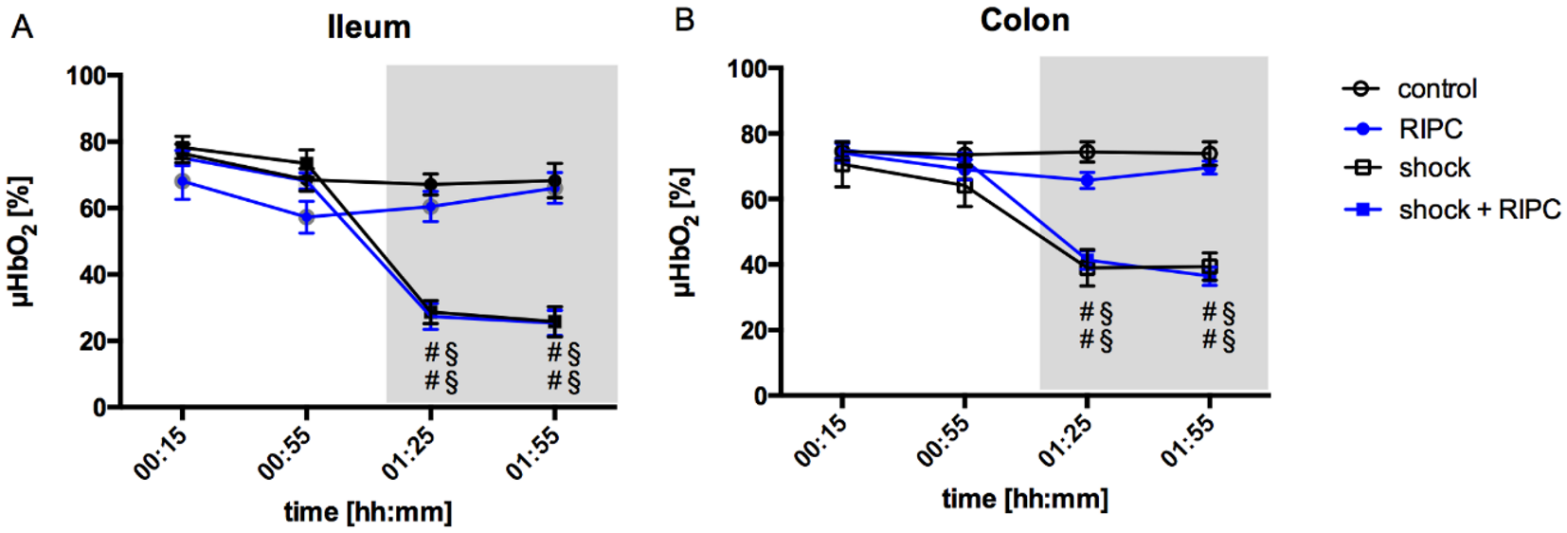
Postcapillary oxygen saturation. Time-related changes of postcapillary oxygen saturation (µHbO_2_) in percentage points (%). Measurements were carried out at outer surface of the terminal ileum (**A**) and the colon ascendens (**B**). Interventional groups received remote ischemic preconditioning treatment (RIPC) before fixed-pressure hemorrhage (shock). Control animals were observed without the induction of remote ischemic preconditioning and/or fixed-pressure hemorrhage. One hour of acute hemorrhage is marked grey. Data are presented as mean ± SEM for n = 12 Wistar-rats. 2-way ANOVA for repeated measurements followed by Bonferroni post-hoc test did not reveal significant differences between experimental groups with or without RIPC treatment under the same hemodynamic conditions. ^#^p ≤ 0.05 vs. baseline values, ^§^p ≤ 0.05 vs. normovolemic control group with the same RIPC-treatment.

Hemorrhagic shock led to a significant reduction of ileal post capillary oxygen saturation (µHbO_2_) from 78 ± 3% to 29 ± 3%. In the simultaneously recorded colonic measurement initial µHbO_2_ values were similar to those of ileum (71 ± 7%) and decreased to 39 ± 6% under otherwise the same hemorrhagic macrovascular conditions. Changes in ileal and colonic µHbO_2_ during hemorrhagic shock were statistically significant compared to their respective normovolemic controls and baseline values. Preinterventional RIPC did not affect ileal (27 ± 4%) and colonic (41 ± 3%) µHbO_2_ during hemorrhagic shock. There was no difference between the experimental groups at baseline levels in the ileum and colon (Fig. 2).

In the terminal ileum microvascular blood flow (µflow) decreased from 181 ± 23 aU to 53 ± 10 aU, when hemorrhage was induced. Colonic µflow decreased from 215 ± 39 aU to 107 ± 37 aU. Whereas results from the initial shock were significantly different to the intragroup baseline, intergroup comparison turned out to be significant in the late phase of hemorrhage. Acute hemorrhage decreased microvascular blood flow velocity (µvelo) and relative hemoglobin content (rHb) compared to the individual baseline value, but RIPC had no effect on intestinal µflow, µvelo and rHb measurements (Table 1).

**Table 1:** Reflectance spectrophotometry and laser Doppler flowmetry.

Variables	Group	00:15 h	00:55 h	01:25 h	01:55 h
Ileal rHb [aU]	Control	82 ± 3	74 ± 3	79 ± 3	76 ± 3
	RIPC	80 ± 3	75 ± 3	79 ± 3	81 ± 2
	Shock	81 ± 2	78 ± 4	62 ± 3^#^	60 ± 3^#§^
	Shock + RIPC	84 ± 2	80 ± 3	56 ± 2^#§^	56 ± 4^#§^
Ileal µflow [aU]	Control	197 ± 28	162 ± 28	166 ± 33	200 ± 40
	RIPC	135 ± 21	146 ± 26	170 ± 33	185 ± 30
	Shock	181 ± 23	148 ± 36	53 ± 10^#§^	71 ± 21^#§^
	Shock + RIPC	159 ± 28	163 ± 36	60 ± 14^#§^	61 ± 16^#§^
Ileal µvelo [aU]	Control	30 ± 3	29 ± 4	28 ± 3	31 ± 4
	RIPC	26 ± 3	26 ± 3	28 ± 3	30 ± 3
	Shock	31 ± 3	24 ± 3	15 ± 1^#§^	15 ± 2^#§^
	Shock + RIPC	28 ± 3	29 ± 3	16 ± 2^#§^	17 ± 2^#§^
Colonic rHb [aU]	Control	88 ± 3	77 ± 4	80 ± 5	78 ± 4
	RIPC	85 ± 4	77 ± 4	76 ± 4	77 ± 4
	Shock	90 ± 2	82 ± 2	64 ± 2^#§^	61 ± 3^#^
	Shock + RIPC	87 ± 2	84 ± 3	64 ± 2^#§^	57 ± 4^#§^
Colonic µflow [aU]	Control	239 ± 37	153 ± 17	228 ± 36	228 ± 57
	RIPC	188 ± 48	195 ± 35	187 ± 35	209 ± 25
	Shock	215 ± 39	160 ± 18	107 ± 37^#^	97 ± 23^#^
	Shock + RIPC	241 ± 45	264 ± 45	104 ± 23^#^	79 ± 17^#^
Colonic µvelo [aU]	Control	29 ± 3	23 ± 1	31 ± 3	28 ± 3
	RIPC	26 ± 4	27 ± 3	27 ± 3	29 ± 3
	Shock	26 ± 3	23 ± 2	16 ± 2^#§^	17 ± 2^#§^
	Shock + RIPC	28 ± 4	30 ± 4	18 ± 2^#^	15 ± 1^#§^

Time-related changes of microcirculatory variables. Data are presented as mean ± SEM for n = 12 Wistar-rats. Statistical significance was accepted for p ≤ 0.05. 2-way ANOVA for repeated measurements followed by Bonferroni post-hoc test did not reveal significant differences between experimental groups with or without RIPC treatment under the same hemodynamic conditions. ^#^p ≤ 0.05 vs. baseline values, ^§^p ≤ 0.05 vs. normovolemic control group with the same RIPC-treatment.

**Figure 3:**
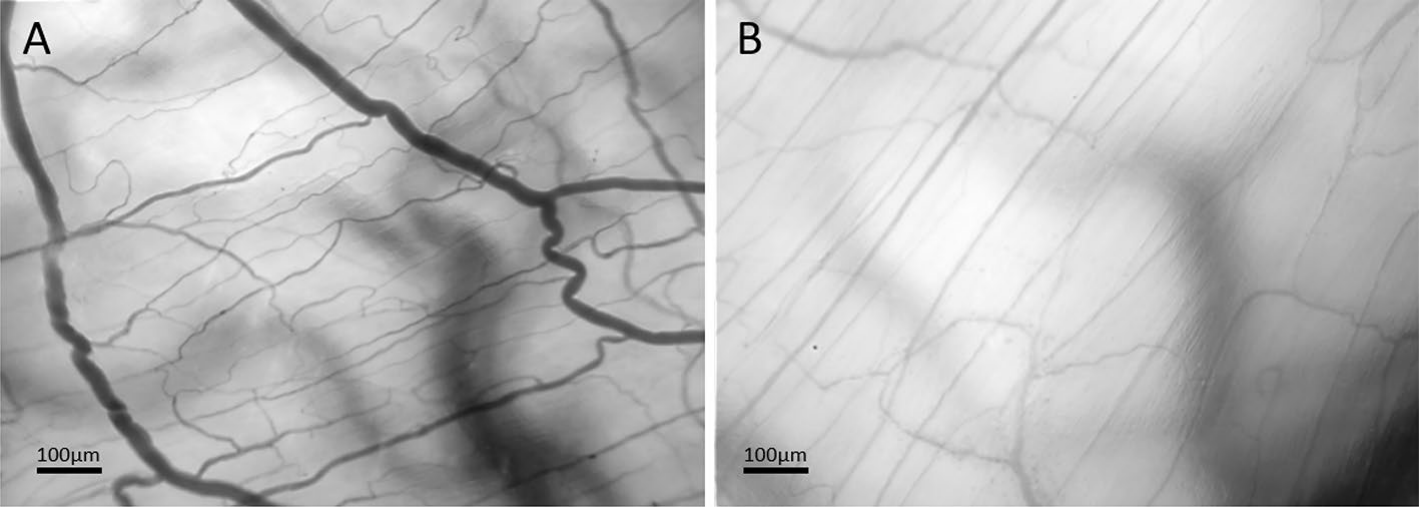
The intestinal microcirculation under physiological conditions and hemorrhagic shock. Visualization of the colonic microcirculation by incident darkfield (IDF) -imaging in the rat (uncorrected magnification factor: 8, field of view: 1.73 mm^2^) under regular hemodynamic conditions (**A**) and hemorrhagic shock (**B**). Red blood cells occur as dark light recesses. Vessels can be detected indirectly by the juxtaposition of red blood cells while passing the microcirculation.

Total vessel density (TVD) and perfused vessel density (PVD) remained unchanged under hemorrhagic conditions. Otherwise, hemorrhage led to a pronounced decrease in microvascular perfusion markers (Fig. 3).

**Figure 4:**
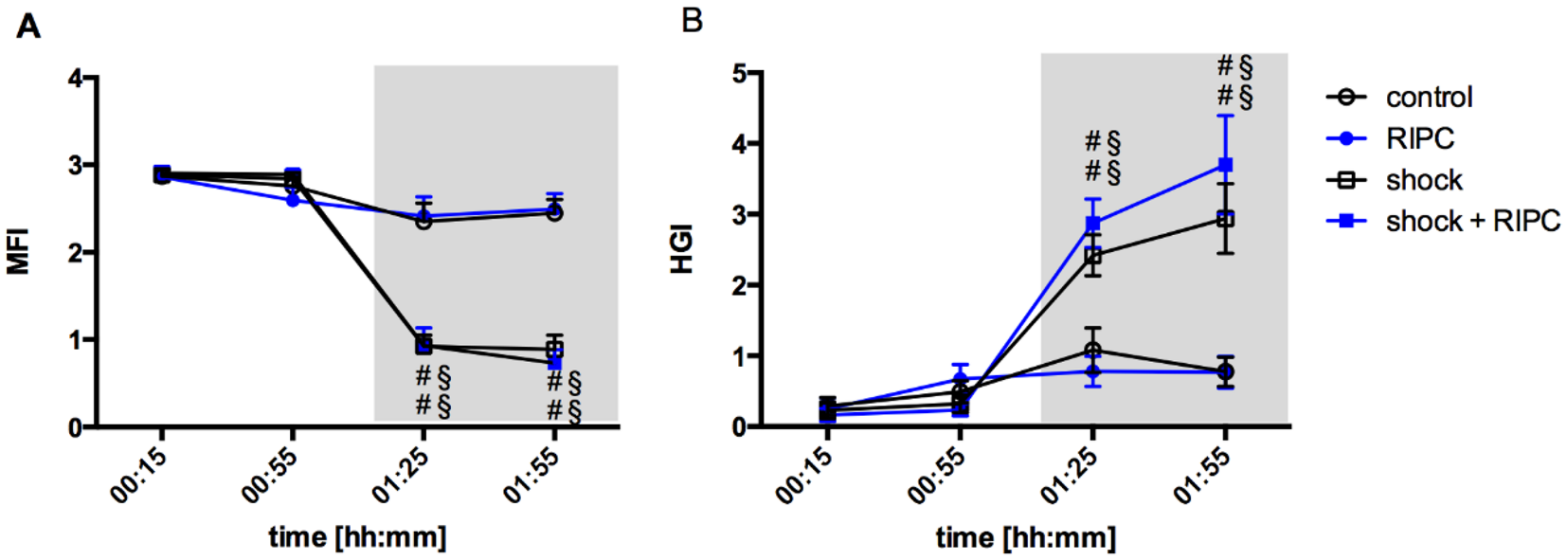
Microvascular flow index and heterogeneity index. Time-related changes of microvascular flow index (MFI, **A**) and heterogeneity index (HGI, **B**). Measurements were carried out at outer surface of the colon ascendens. Interventional groups received remote ischemic preconditioning treatment (RIPC) and/or fixed-pressure hemorrhage (shock). Control animals were observed without the induction of remote ischemic preconditioning and/or fixed-pressure hemorrhage. One hour of acute hemorrhage is marked grey. Data are presented as mean ± SEM for n = 12 Wistar-rats. 2-way ANOVA for repeated measurements followed by Bonferroni post-hoc test did not reveal significant differences between experimental groups with or without RIPC treatment under the same hemodynamic conditions. ^#^p ≤ 0.05 vs. baseline values, ^§^p ≤ 0.05 vs. normovolemic control group with the same RIPC-treatment.

In detail, microvascular flow index (MFI) decreased from 2.9 ± 0.1 to 0.9 ± 0.1 in the shock group. Shock animals after RIPC had similar MFI-values, which decreased from 2.9 ± 0.1 to 0.9 ± 0.0. There was no difference between hemorrhagic animals with or without RIPC-treatment. Normovolemic animals did not show any significant alterations of MFI. Further, heterogeneity index (HGI) increased in animals with hemorrhage and reached values of 2.4 ± 0.3 in the early and 2.9 ± 0.5 in the late course of hemorrhagic shock (baseline: 0.2 ± 0.1). There was no effect of RIPC pretreatment on HGI (Fig. 4) (Table 2).

**Table 2:** Videomicroscopy.

Variables	Group	00:15 h	00:55 h	01:25 h	01:55 h
TVD [mm/mm^2^]	Control	14 ± 1	13 ± 2	13 ± 1	12 ± 1
	RIPC	13 ± 1	12 ± 1	13 ± 1	14 ± 1
	Shock	13 ± 1	13 ± 1	10 ± 2^#^	11 ± 1
	Shock + RIPC	11 ± 1	11 ± 0	9 ± 1	11 ± 1
PVD [mm/mm^2^]	Control	6 ± 1	6 ± 1	6 ± 0	6 ± 1
	RIPC	5 ± 1	4 ± 1	6 ± 1	7 ± 1^#^
	Shock	5 ± 1	6 ± 1	3 ± 1^§^	4 ± 0
	Shock + RIPC	4 ± 0	4 ± 0	4 ± 1	3 ± 1^§^

Time-related changes of microcirculatory variables. Data are presented as mean ± SEM for n = 12 Wistar-rats. Statistical significance was accepted for p ≤ 0.05. 2-way ANOVA for repeated measurements followed by Bonferroni post-hoc test did not reveal significant differences between experimental groups with or without RIPC treatment under the same hemodynamic conditions. ^#^p ≤ 0.05 vs. baseline values, ^§^p ≤ 0.05 vs. normovolemic control group with the same RIPC-treatment.

### Mitochondrial respiration and oxidative stress

**Figure 5:**
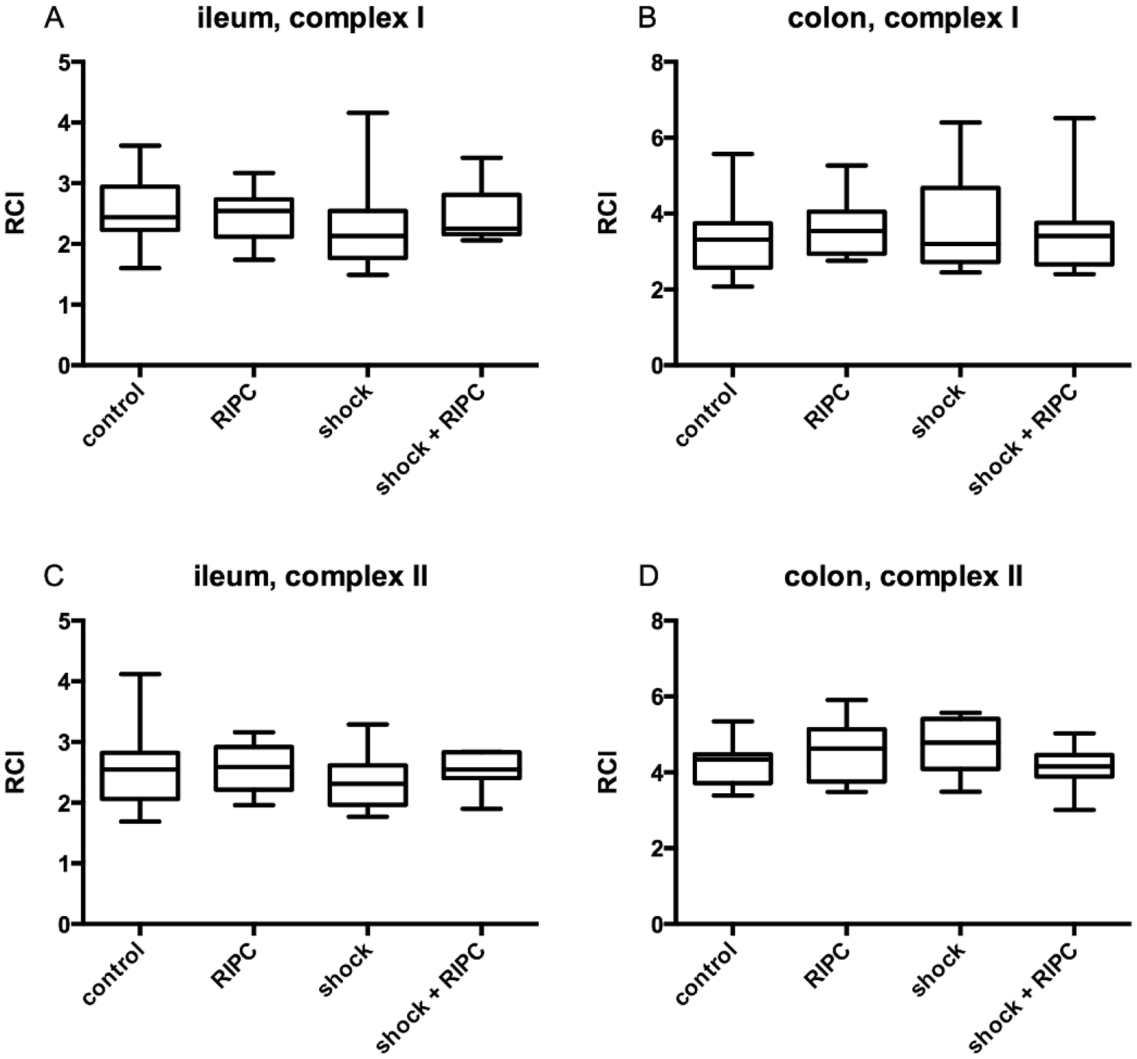
Respiratory control index. Mitochondrial coupling in ileal and colonic tissue homogenates were determined by the respiratory control index (RCI) after separated stimulation of complex I (glutamate and malate; **A**,**B**) and complex II (succinate; **C**,**D**). Remote ischemic preconditioning (RIPC) and/or a fixed-pressure shock was induced in vivo before the determination of RCI. Control animals received general anesthesia and standardized instrumentation without RIPC and/or shock. Data are presented for n = 12 as median and interquartile range. Whiskers indicate maximal and minimal values. Kruskal–Wallis testing and Dunn’s multiple comparison did not reveal significant differences between the experimental groups.

**Figure 6:**
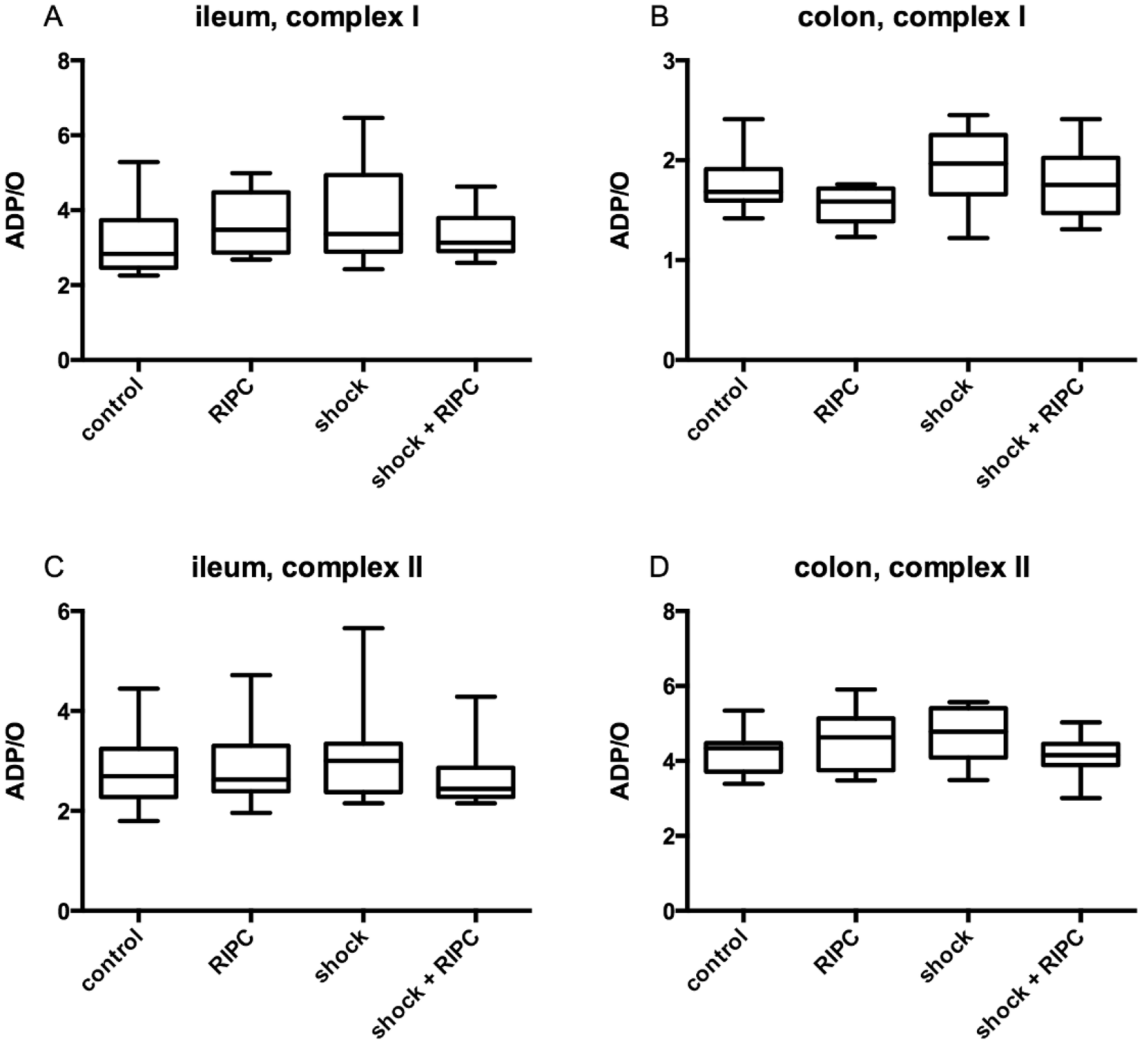
ADP/O-ratio. Efficiency of oxidative phosphorylation in ileal and colonic tissue homogenates was determined by measurement of ADP/O-ratio after separated stimulation of complex I (glutamate and malate; **A**,**B**) and complex II (succinate; **C**,**D**). Remote ischemic preconditioning (RIPC) and/or a fixed-pressure shock was induced in vivo before the determination of ADP/O-ratio. Control animals received general anesthesia and standardized instrumentation without RIPC and/or shock. Data are presented for n = 12 as median and interquartile range. Whiskers indicate maximal and minimal values. Kruskal–Wallis testing and Dunn’s multiple comparison did not reveal significant differences between the experimental groups.

In tissue homogenates of both, ileum and colon, respiratory control index (RCI) was measured to determine mitochondrial coupling between the electron transfer system and the oxidative phosphorylation (Fig. 5). Further, ADP/O-ratio was measured to mirror the efficiency of the oxidative phosphorylation (Fig. 6). In control animals after complex I stimulation RCI values of 2.44 ± 0.71 in ileum samples (Fig. 5A) and 3.32 ± 1.17 in the colon (Fig. 5B) were reached. If complex II was stimulated, RCI was 2.55 ± 0.76 in ileum (Fig. 5C) and 4.35 ± 0.76 in colon (Fig. 5D). Neither shock induction nor RIPC-treatment influenced RCI.

ADP/O-ratio was 2.84 ± 1.27 in ileum samples and 1.69 ± 0.32 in colon samples after complex I stimulation (Fig. 6A,B). Values did not differ significantly after addition of succinate as complex II substrate (Fig. 6C,D). During acute hemorrhage mitochondrial function was maintained in ileum and colon tissue. RIPC affected mitochondrial respiration neither under physiological conditions nor during hemorrhage compared to the respective control group.

There were no alterations of electron transfer within the respiratory chain and the maximal mitochondrial respiration between normovolemic controls and interventional groups (Table 3).

**Table 3:** Mitochondrial respiration.

	Groups	State 2	State 3
Ileum complex I [nmol·min^−1^·mg^−1^]	Control	0.39 ± 0.08	1.01 ± 0.27
	RIPC	0.43 ± 0.18	1.01 ± 0.23
	Shock	0.38 ± 0.11	0.85 ± 0.38
	Shock + RIPC	0.38 ± 0.09	0.96 ± 0.24
Ileum complex II [nmol·min^−1^·mg^−1^]	Control	0.54 ± 0.16	1.21 ± 0.43
	RIPC	0.49 ± 0.09	1.25 ± 0.29
	Shock	0.51 ± 0.18	1.18 ± 0.41
	Shock + RIPC	0.47 ± 0.08	1.3 ± 0.32
Colon complex I [nmol·min^−1^·mg^−1^]	Control	0.49 ± 0.21	1.54 ± 0.33
	RIPC	0.53 ± 0.17	1.78 ± 0.37
	Shock	0.43 ± 0.03	1.48 ± 0.46
	Shock + RIPC	0.53 ± 0.11	1.65 ± 0.48
Colon complex II [nmol·min^−1^·mg^−1^]	Control	0.65 ± 0.13	2.74 ± 0.49
	RIPC	0.71 ± 0.19	3.08 ± 0.7
	Shock	0.6 ± 0.115	2.93 ± 0.53
	Shock + RIPC	0.66 ± 0.14	2.76 ± 0.55

Electron transfer (state 2) and maximal mitochondrial respiration (state 3) measured respirometry in ileal and colonic tissue homogenates. Data are presented as median ± interquartile range for n = 12 Wistar-rats. Statistical significance was accepted for p ≤ 0.05. Kruskal–Wallis testing and Dunn’s multiple comparison did not reveal significant differences between experimental groups with or without RIPC treatment under the same hemodynamic conditions.

**Figure 7:**
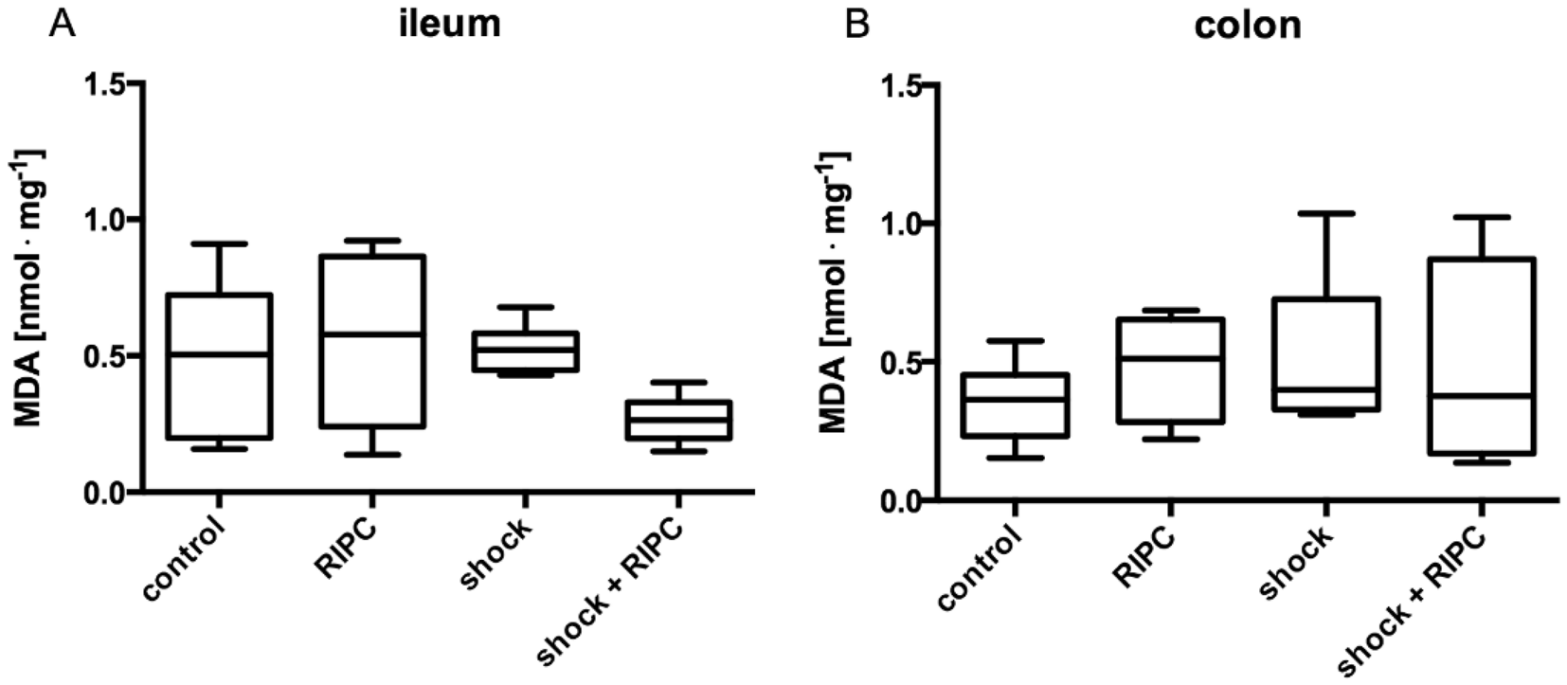
Malondialdehyde concentration. Lipid peroxidation was determined by Malondialdehyde (MDA) concentration in nmol/mg protein of ileal and colonic tissue homogenates. Remote ischemic preconditioning (RIPC) and/or a fixed-pressure shock was induced in vivo before the determination of MDA concentration. Control animals received general anesthesia and standardized instrumentation without RIPC and/or shock. Data are presented for n = 12 as median and interquartile range. Whiskers indicate maximal and minimal values. Kruskal–Wallis testing and Dunn’s multiple comparison did not reveal significant differences between the experimental groups.

Malondialdehyde (MDA) concentration of controls without RIPC intervention were 0.5 ± 0.53 nmol · mg^−1^ in ileal samples and 0.36 ± 0.22 nmol · mg^−1^ in colonic tissue. Pretreatment with RIPC and the induction of acute hemorrhagic shock did not alter intestinal MDA concentrations (Fig. 7).

### Intestinal tissue integrity

#### Plasmatic d-lactate concentration

**Figure 8:**
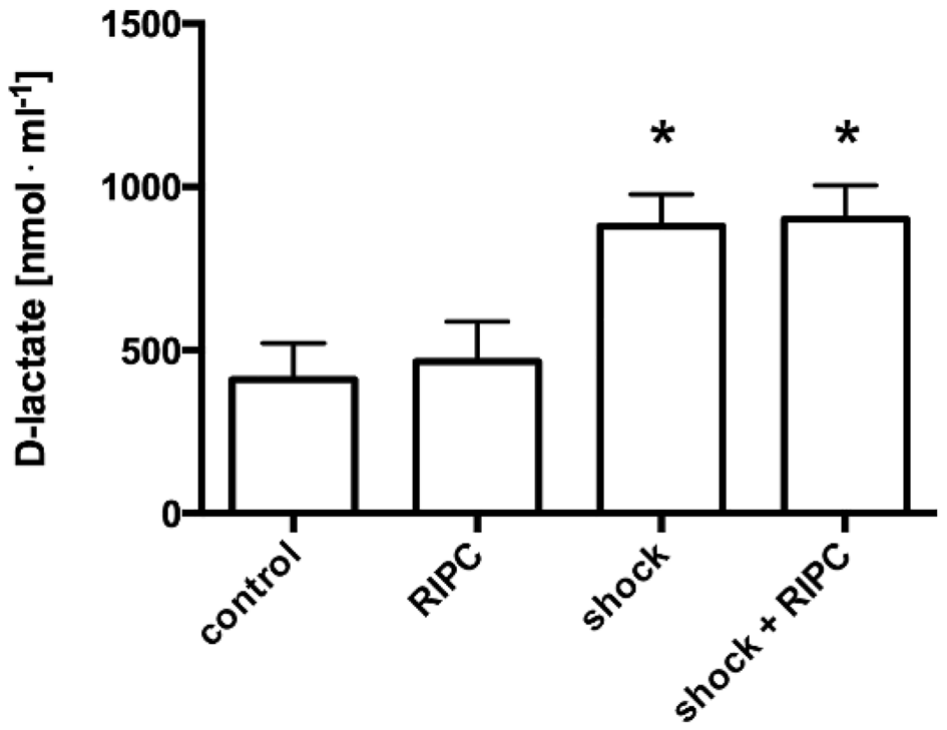
Plasmatic d-lactate concentration. Intestinal barrier function was determined by plasmatic d-lactate concentrations in nmol/ml plasma volume after the termination of the experiments. Interventional groups received remote ischemic preconditioning treatment (RIPC) and/or fixed-pressure hemorrhage (shock) in vivo. Control animals were observed without the induction of remote ischemic preconditioning and/or fixed-pressure hemorrhage. Data are presented as mean ± SEM for n = 12 Wistar-rats. One way-ANOVA and Sidak post-hoc correction for multiple comparison did not reveal significant differences between experimental groups with or without RIPC treatment under the same hemodynamic conditions. *p < 0.05 vs. normovolemic group.

d-Lactate concentrations were 409 ± 112 nmol · ml^−1^ plasma in control animals and 464 ± 122 nmol · ml^−1^ plasma in animals with RIPC-treatment. Plasmatic d-lactate concentrations in hemorrhagic animals were significantly increased in shock animals (shock: 880 ± 98 nmol · ml^−1^ plasma). RIPC did not reduce plasmatic d-lactate levels (shock + RIPC: 900 ± 104 nmol · ml^−1^ plasma) (Fig. 8).

#### Histology

Histological slides showed no signs of tissue injury in animals with hemorrhagic shock. Further, there was no effect of RIPC on Chiu-score values (data not shown).

## Discussion

This study was designed to characterize intestinal damage during acute hemorrhage and to investigate if RIPC is able to improve the selected variables in a rodent model of hemorrhagic shock. The here obtained data of microcirculatory and mitochondrial analyses supplemented by markers of structural and functional intestinal impairment lead to the following main findings.

(i) In a model of fixed-pressure hemorrhage postcapillary oxygen saturation and microvascular flow index seem to be sensitive markers to detect microvascular impairment, whereas microcirculatory vessel architecture is maintained.
(ii) Mitochondrial respiration is not affected during early hemorrhagic shock states.
(iii) Plasmatic d-lactate levels but not histological injury markers increase significantly after 1 h of hemorrhagic shock.
(iv) Remote ischemic preconditioning neither improves microcirculatory variables nor plasmatic d-lactate levels during hemorrhagic shock.

During the past decades a deeper understanding of the microvascular compartment provoked the acceptance of the microcirculation as an independent functional unit of the cardiovascular system. Microvascular vessels might react differently from macrocirculatory hemodynamics to meet the individual needs of the corresponding tissue, a concept termed hemodynamic incoherence^22^. Especially in critically ill patients, physicians suspected for years the existence of hemodynamic incoherence from clinical expertise. However, medical treatment relied on indirect variables to monitor microvascular oxygen delivery, tissue perfusion and cell integrity. Therefore, the invention and development of devices thought to investigate microcirculation directly were a cornerstone of modern intensive care research^23^. Trzeciak et al. reported an improvement of microvascular flow in septic patients to be associated with a decrease in multi-organ failure after 24 h despite the registration of similar macrocirculatory variables^24^. Further, the persistence of microcirculatory impairment was associated with higher mortality, regardless of whether therapeutic approaches were able to resolve severe shock states or not^25^. Since microvascular variables turned out to be more sensitive than macrocirculatory hemodynamics to predict outcome in critical illness, microcirculatory variables are already discussed to be implemented into clinical decision-making. Most findings of microvascular alterations in so-called “critical illness” are derived from septic patient collectives. Only a few clinical trials were undertaken to strengthen the assumption that microcirculatory aberrations in hemorrhagic patients are able to predict outcome similar to findings in septic patients^26,27^. In both trials perfused vessel density and microvascular flow index as variables of microvascular perfusion turned out to mirror hemorrhage, whereas total vessel density as a marker for microvascular architecture did not change over the time. Those findings are in accordance with previous observations from our working group^28^. Finally, perfusion markers are reproducibly capable for microvascular monitoring in hemorrhagic sheep, dogs and rats and independent of the modus of shock induction^23^. In 2018 an expert group from the Cardiovascular Dynamics Section of the European Society of Intensive Care Medicine published a second consensus paper with recommendation how to evaluate the microcirculation in critically ill patients^29^. Based on the listed publications the MFI and TVD are considered as first choice variables, which should be reported in trials focusing on hemorrhagic patients. Our results extend the body of literature indicating MFI as a sensitive variable to indicate early microvascular changes during hemorrhagic shock in animal testing. Therefore, we recommend the reporting of MFI and TVD not only in clinical trials but also in hemorrhagic shock animal models. This study combines microvascular evaluation by IDF-imaging with the determination of postcapillary oxygen saturation as a marker of local oxygen reserve. Our data implicate, that an impairment of microvascular blood flow results in a decrease of postcapillary oxygen saturation during hemorrhagic shock without alterations of microvascular architecture. In detail, reduction of local blood flow is caused by a decrease of blood flow continuity and spatial blood flow heterogeneity determined by IDF-imaging as well as total microvascular blood volume movement measured by laser-Doppler in relative units. Boerma et al. reported a correlation of sublingual and intestinal microcirculation in states of microcirculatory derangements^30^. In consequence, sublingual microcirculation as a non-invasively accessible region was recommended for microcirculatory assessment in critically ill patients^29^. Interestingly, previous data of our group indicate, that the protective effect of local carbon dioxide application on mucosal oxygenation differs in a time-related manner between oral and gastral mucosa^28^. Therefore, the transferability of indices of the microcirculation obtained at one specific side should be considered critically. However, our results highlight the importance of monitoring hemodynamic incoherence in severe shock states and suggest, that microvascular monitoring by IDF-imaging as a current state of the art method might profit from an additional metabolic evaluation to detect tissues at risk for ischemic injury.

Mitochondrial impairment and reactive oxygen species were suggested to be responsible for aggravating and multiplying tissue injury in states of restricted oxygen delivery^31^. In our experiments electron transfer within the respiratory chain, maximal respiration rate, mitochondrial coupling and the respiratory efficiency were maintained after 1 h of hemorrhagic shock and did not differ between normovolemic controls and the hemorrhagic interventional group. Mitochondrial function was reported to act independent of metabolism in other cell components^32^. It seems reasonable that mitochondrial dysfunction occurs late during shock associated cell damage, since mitochondria are the structural correlate of indispensable cellular energy generation. In conclusion, protection of mitochondrial integrity is essential for the reestablishment of physiological cell function after adverse events and cell damage might manifest earlier in other cell compartments. Oxidative stress might impair cell integrity and lead to further functional impairment of stroma and endothelial cells under adverse conditions^33^. However, intestinal MDA concentration did not differ between animals receiving hemorrhagic shock and animals under physiological conditions. There is a lack in a simultaneously reporting of microvascular and mitochondrial variables to reveal time-related pathophysiological changes during early hemorrhage^34^. According to the findings from this study microcirculatory oxygen supply is reduced early during hemorrhagic shock, whereas mitochondrial respiration is unchanged after 1 h of hemorrhage. Therefore, we assume microvascular derangements rather than mitochondrial failure to be responsible for functional tissue damage during early hemorrhagic shock. This might change in models of prolonged shock with suspected further energy depletion. Further, vascular reperfusion could induce secondary mitochondrial failure in consequence to an increased load of reactive oxygen species and proinflammatory mediators. Since mitochondrial failure was observed after intestinal ischemia–reperfusion injury^35^, further studies should address if prolonged shock states and/or the reperfusion phase induce mitochondrial failure in intestinal tissue after prolonged shock states and ischemia reperfusion injury.

The present study was designed to relate alterations of postcapillary oxygen saturation and microvascular flow index, as the most sensitive variables to detect microvascular impairment with markers of impaired tissue integrity. Plasmatic d-lactate concentrations were reported to mirror intestinal damage after severe injuries. In a comparative study on male Wistar rats, total gut ischemia by mesenteric artery clamping as well as severe burn injury and acute necrotizing pancreatitis led to a significant increase of plasmatic d-lactate concentrations^36^. d-Lactate levels from individuals receiving total ischemia raised after 1 h of ischemia. Both, total ischemia and severe hemorrhage are characterized by a failure of microvascular oxygen supply as a result of blood flow restriction. Nevertheless, pathophysiology is different especially in the microcirculatory compartment since residual blood flow occurs only in hemorrhagic animals. However, plasmatic d-lactate levels increased significantly in hemorrhagic animals compared to normovolemic individuals after 1 h of hemorrhagic shock in our experiments. These results are in accordance with previous findings from Szalay et al. reporting increased plasma levels of d-lactate in an experimental animal model of hemorrhagic shock^37^. In a single-center, observational, prospective trial Li et al. could indicate, that plasmatic d-lactate concentration was associated with the grade of acute gastrointestinal injury^38^ according to the definition of the European Society of Intensive Care Medicine in critically ill patients. In this trial d-lactate concentration served as a better marker of gastrointestinal failure than lipopolysaccharide and levels of intestinal fatty acid-binding protein. Summing up, plasmatic d-lactate concentration is a marker of gastrointestinal damage and functional impairment during hemorrhagic shock. In this study microvascular derangements were associated with an increase of d-lactate concentrations during hemorrhagic shock. Since higher d-lactate concentrations are related to acute gastrointestinal failure and mortality scores in critically ill patients, early monitoring of microcirculatory variables and plasmatic d-lactate concentrations could identify patients at risk to develop secondary complications after hemorrhagic shock. During hemorrhagic shock we could observe severe macroscopic changes of the intestine. However histological evaluation failed to mirror intestinal damage after hemorrhagic shock in our experiments. Since the Chiu scoring system revealed reliable and reproducible results despite its simple application in experimental practice^39^, there might be three reasons for this finding. First, it is doubtful if tissue injury during hypoxic states is caused exclusively by the hypoxic stimulus. In models of total ischemia, it is well described that not only the ischemic event but the reperfusion itself leads to severe tissue injury. This finding is called ischemia–reperfusion injury and occurs when acidic agents and reactive oxygen species accumulate during hypoxia^40^. Reperfusion injury could lead to additional tissue damage and a histologically visible impairment of intestinal integrity. However, the aim of the here presented study was to describe the effect of isolated hemorrhagic shock on intestinal integrity. Second, the lack of intestinal damage in histological slides could be a time related phenomenon. Histological injury after ischemia is well described in models of myocardial infarction and follows a characteristic time-related pattern^41^. Despite the extensive energy metabolism of myocardial cells, histological changes occur after several hours. In fact, many studies investigating the effect of remote ischemic preconditioning on intestinal damage terminated the experimental protocol after 24 h^18,19^. In the present study a further delay between shock and tissue sample acquisition was not possible due to the invasive instrumentation including arterial and venous catheterization and surgical airway management. We conclude, that plasmatic d-lactate concentration but not histological stainings are suitable to detect early injury of the intestine. Third, the inflammatory response to trauma and hemorrhage is a field of rising interest^42^. A recent study reported an activation of the immune system with subsequent cytokine release in experimental models of shock and trauma^43^. Since systemic and local inflammation is linked to tissue injury and barrier dysfunction^44^, the divergence between histological integrity and functional impairment could be a result of cytokine related barrier dysfunction^45^. Especially in the gastrointestinal tract, epithelial barrier function is closely linked to a complex system of adherence proteins^46^. These molecular formations can be visualized by more accurate methods like electron transmission microscopy that could link impairment of intestinal functionality to ultrastructural damage of intestinal cells. Further studies should clarify, if electron transmission microscopy could reveal more distinct intestinal damage after isolated hemorrhagic shock or after combined hemorrhage-reperfusion.

Finally, this study was designed to investigate the effects of RIPC on intestinal microcirculation, mitochondrial function, and tissue integrity. However, microcirculatory variables and tissue integrity did not improve in our experiments after RIPC-treatment. Only a few studies were designed to clarify the impact of RIPC on microcirculatory variables before. Kraemer et al. used a combined Laser-Doppler and photospectrometry measurement to investigate the remote effect of ischemic preconditioning on cutaneous microcirculation in healthy subjects. Cutaneous oxygen saturation and microvascular blood flow increased significantly after RIPC-treatment whereas postcapillary venous filling pressure decreased compared to baseline values^47^. Further, limb ischemic preconditioning also exerts a protective effect on abdominal microcirculation. In a model of chronic kidney injury in rats RIPC was able to improve renal medullary blood flow and oxygenation^48^. Those findings are derived from healthy or chronically ill individuals. Pathophysiology might be different in individuals with acute adverse events since adaptive mechanisms have not been established further. However, Tapuria et al. stated a beneficial effect of RIPC on microcirculatory variables in a model of acute hepatic ischemia–reperfusion injury, too^49^. Finally, Koike et al. transferred the concept of RIPC in a model of necrotizing enterocolitis in rats with a beneficial effect of pretreatment on intestinal blood flow velocity^50^. Those findings diverging to ours are most likely not a result of interspecies differences and the use of different preconditioning regimes since all cited publications of abdominal microcirculatory protection used the same RIPC-treatment in rodent animal models. Further, there is no evidence that the success of RIPC-treatment in the rodent intestine is dose-dependent with extended RIPC-regimes related to a higher grade of tissue protection^51^.

The inconstant reporting of RIPC related tissue protection led to the identification of various confounders which might diminish the beneficial effects of RIPC in experimental testing and might explain, why the concept of RIPC was not transferred successfully to clinical trials. Since commonly used anesthetics and analgetic substances were reported to interfere with RIPC^52^, we used a mono-drug pentobarbital anesthesia regime in our experiments. Furthermore, only male animals were used to minimize the effect of endocrinological differences between male and female rats on the physiological responses to trauma and RIPC^53^. Despite the high grade of standardization in our experimental protocol, tissue protection by RIPC could be abolished by not-addressed variables. First, we aimed to induce a severe hemorrhagic shock and targeted a fixed-pressure shock with mean arterial blood pressure levels of about 40 ± 5 mmHg. A decrease of mean arterial blood pressure of approximately 90 mmHg, although 70% of the registered baseline value, might represent a microcirculatory shock with a failure of autogenous compensatory mechanisms. On the one hand RIPC therefore might exert its beneficial effects on intestinal microcirculation under conditions of milder hemorrhagic shock. On the other hand, nearly all studies that reported a beneficial effect of RIPC on tissue damage used models of ischemia–reperfusion injury. In fact, pathophysiology differs between ischemia and hemorrhage with a persistent microvascular blood flow during hemorrhagic shock and a complete interruption of microvascular oxygen supply in models of ischemia–reperfusion injury. This might also explain the divergence between studies that recently reported a beneficial effect of RIPC on intestinal damage after complete vascular occlusion and this data set^18,19^. Therefore, it is also possible that the hypoxic stimulus during hemorrhage is insufficient to reveal the protective effect of RIPC. Further studies should elucidate the impact of different hemodynamic conditions on RIPC-dependent tissue protection. In addition, commonly used models of ischemia–reperfusion injury do not only implicate a period of restricted oxygen supply, but also a period of reperfusion^31,35^. According to the concept of reperfusion injury, the phase of tissue reperfusion can not only be related to additional tissue damage, but also to a further potential of tissue protection by RIPC. In the same way, protective mechanisms of RIPC frequently become apparent about one day after the RIPC-treatment called “the late window of tissue protection”. Investigators should focus on RIPC-derived tissue protection during blood reperfusion and the late window of tissue protection.

## Conclusion

The intestinal microcirculation is affected severely when hemorrhagic shock occurs. According to recommendations of the ESICM for microvascular evaluation in humans, microvascular perfusion markers and especially the microvascular flow index can detect microvascular impairment early during hemorrhagic states and should also be determined in animal models of hemorrhagic shock. Whereas microcirculatory architecture is generally maintained in the intestine, postcapillary oxygen saturation decreases immediately after the induction of hemorrhage and might implicate a functional aspect of microvascular evaluation. According to our knowledge, only a few studies have measured microcirculation and mitochondrial function during hemorrhage in the same animals. Surprisingly, mitochondrial integrity is generally maintained in this model of experimental hemorrhage and does not seem to be impaired early in the course of acute hemorrhage. We state that microvascular failure is the predominant mechanism of hemorrhage associated tissue injury highlighting the need to establish microvascular evaluation especially in patients with trauma or hemorrhagic shock. Plasmatic d-lactate levels mirror the early loss of intestinal barrier integrity whereas histological preparations are not sensitive enough to detect early intestinal tissue damage after 1 h of severe hemorrhage. This emphasizes the role of plasmatic d-lactate concentration to monitor intestinal injury especially during hemorrhagic shock.

RIPC is frequently described to improve microcirculatory variables and tissue integrity in models of ischemia reperfusion. In our experiments RIPC did not exert beneficial effects on tissue oxygenation, microvascular perfusion and markers of intestinal tissue integrity in the context of a severe hemorrhagic shock. This might be the effect of a prostrated autogenous compensation during severe shock states. Further investigation should clarify the impact of the hemodynamic context and early tissue reperfusion on RIPC-mediated tissue protection in the gastrointestinal tract. Finally, uncovering the molecular mechanisms could enable a successful transfer of preclinical findings into medical treatment.

## Methods

### Animals and instrumentation

All experiments were performed in accordance with guidelines of the national institute of health guidelines for animal care and approved from the local Animal Care and Use Committee (Landesamt für Natur, Umwelt und Verbraucherschutz, Recklinghausen, Germany, AZ. 81-02.04.2018.A308). The study follows the ARRIVE guidelines. Animals were bred for experimental purposes and derived from the animal research facility of the Heinrich-Heine-University Duesseldorf (ZETT, Zentrale Einrichtung für Tierforschung und wissenschaftliche Tierschutzaufgaben).

A total of 48 male Wistar rats (350 ± 35 g body weight) were randomized into four groups with 12 individuals each (n = 12). All animals received anesthesia (sodium pentobarbital 100 mg·kg^−1^ i.p. as bolus followed by 10 mg·kg^−1^·h^−1^ i.v.) and standardized instrumentation via cervical surgical access consisting of tracheostomy, an invasive arterial blood pressure measurement and a central venous catheterization. Adequate anesthesia was verified by testing involuntarily defensive reactions on successive pain stimuli before instrumentation. Previously, we reported carbon dioxide to improve microvascular variables in a model of fixed-volume hemorrhagic shock in dogs^28^. Therefore, ventilation was adjusted to normocapnic and normoxic ventilation by intermittent arterial blood gas analysis. Likewise, rectal temperature was measured to determine central body temperature and animals were placed on heating plates to exclude mild hypothermia as a confounding factor^54^. A median laparotomy was performed to enable a direct assessment of postcapillary oxygen saturation and microcirculatory variables. The ileocecal junction was gently exposed for continuous and intermittent microcirculatory assessment. Finally, blood pressure cuffs were placed around both hind limbs to induce RIPC.

### Experimental protocol

**Figure 9:**
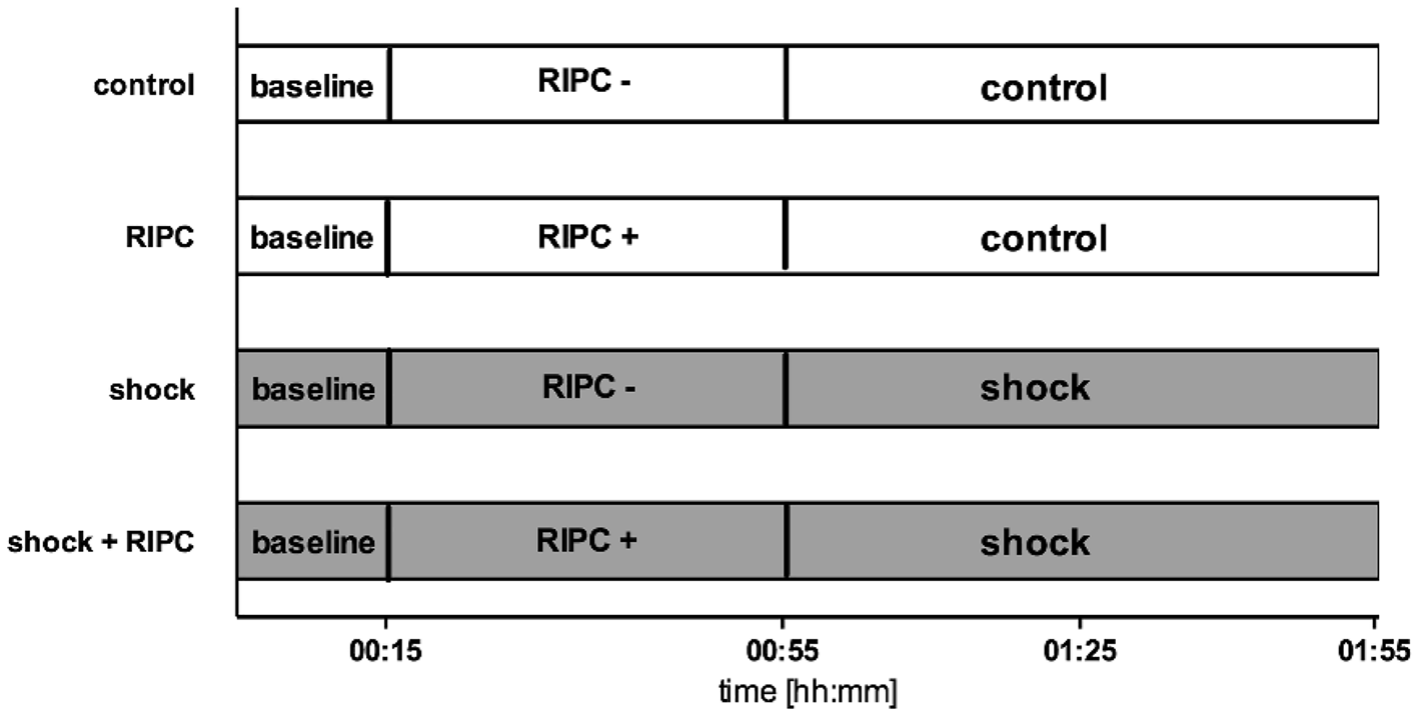
Experimental protocol. The shock + RIPC group received remote ischemic preconditioning treatment (RIPC+) before fixed-pressure hemorrhage (shock). In the shock group acute hemorrhage was performed without prior induction of remote ischemic preconditioning (RIPC−). The RIPC group received remote ischemic preconditioning under otherwise physiological hemodynamic conditions (control). Individuals in the control group were observed without the induction of remote ischemic preconditioning (RIPC-) or fixed-pressure hemorrhage (control).

After standardized instrumentation, animals were observed for at least 15 min to verify steady-state conditions. Steady-state conditions were defined as the stability of hemodynamic and microcirculatory variables for more than 10 min. Baseline values were recorded and animals were randomized to one of the four experimental groups: (1) control, (2) RIPC, (3) shock and (4) shock + RIPC (Fig. 9). In animals that received RIPC, both hind limb cuffs were insufflated to 200 mmHg for 5 min followed by 5 min deflation. This protocol was repeated 4 times. Control animals did not receive remote ischemic preconditioning, while anesthesia was continued for 40 min. Then, a fixed-pressure hemorrhage was induced by removing arterial blood via the arterial catheter, until a mean arterial blood pressure of 40 ± 5 mmHg was reached. In the case of hemodynamic stabilization by endogenous compensatory mechanisms, intermittent blood withdrawal was performed to maintain a constant mean arterial blood pressure. After a shock duration of 1 h, experiments were terminated. In control animals, experiments were continued for 1 h without induction of hemorrhage. All animals were sacrificed after the experiments in deep anesthesia (additional injection of 15 mg · kg^−1^ i.v. pentobarbital) by exsanguination via the arterial catheter. Ileal and colonic tissue as well as blood samples were stored at − 80 °C for further analysis.

### Microcirculatory measurements

#### Reflectance spectrophotometry and laser Doppler flowmetry

Postcapillary oxygen saturation (µHbO_2_) and relative hemoglobin amount (rHb) were assessed by tissue reflectance spectrophotometry, whereas microvascular blood flow amount (µflow) and velocity (µvelo) were determined by laser Doppler flowmetry (O2C, LEA Medizintechnik, Gießen, Germany).

In detail, the measuring probes (Flat Probe LFX-2, LEA Medizintechnik GmbH, Gießen, Germany) were placed on the outer surface of the terminal ileum and the colon ascendens. White light (450–1000 nm) is emitted into the tissue, scattered at cellular components like mitochondria^55^ and absorbed by hemoglobin. Since hemoglobin absorbs different wavelengths of white light depending on its oxygen saturation grade^56^, the averaged hemoglobin saturation can be calculated from changes in the spectral components of the reflected light. Blood is not distributed equally within microcirculatory components, with the highest amount of hemoglobin molecules in the postcapillary section, serving as a capacity compartment. Therefore, tissue reflectance spectrophotometry mainly determines postcapillary oxygen saturation due to its inability to distinguish between arterial and venous vessels within the targeted tissue. Summing up, µHbO_2_ is a sensitive marker to assess metabolic oxygen dept and local oxygen reserve. Relative hemoglobin amount can be calculated from all over signal of light reflection.

Further, a diode-generated pulse laser (CW-mode) with near-infrared wavelength (820 nm) is used to provoke a frequency shift at the surface of moving particles like erythrocytes. According to the Doppler shift principle, red blood cell velocity and microvascular blood flow can be calculated^57^. Both measurements, the tissue reflectance spectrophotometry as well as the laser Doppler flowmetry, were performed as a combined measurement with a continuous assessment. Since vessels larger than 100 µm lead to total light absorption, measurements mirror microvascular conditions^58^. All values are reported as 5 min means under steady-state conditions.

#### Videomicroscopy

Videomicroscopy was used to visualize the microcirculation at the initial part of the colon ascendens intermittently. Incident dark field (IDF)-imaging (CytoCam, Braedius Medical, Huizen, The Netherlands) represents the third-generation handheld videomicroscope^59^. Technically, a complex lens system is surrounded by laser diodes. Pulsed green light of 530 nm wavelength, the isosbestic point of oxygenated and deoxygenated hemoglobin^60^ is emitted to the tissue of interest. Whereas interstitial cells and matrix components are leading to light reflection, erythrocytes as the main hemoglobin carriers absorb the emitted light and can be registered as dark light recesses by the camera.

The resulting videos were saved anonymized for blinded analysis. Only videos that fulfilled the requirements recently defined by an international expert round^61^ were included in this study. The microcirculatory flow index (MFI), a well-established score to access the predominant microvascular blood flow characteristics, was used to indicate microvascular blood flow as “not existent” (scoring = 0), “intermittent flow” (scoring = 1), “sluggish flow” (scoring = 2) or “regular blood flow” (scoring = 3). Regional differences of MFI-values were used to calculate the heterogeneity index (HGI) as a marker of spatial blood flow heterogeneity. Further analysis using the appropriate software (MicroCirculation Analysis software, Braedius Medical, Huizen Netherlands) was provided to determine the total vessel density (TVD) and the perfused vessel density (PVD). All videos were recorded by the same investigator and in close vicinity to O_2_C-measurements to enable a complimentary consideration of IDF-videos, reflectance spectrophotometry and laser Doppler flowmetry.

### Measurement of mitochondrial respiration and oxidative stress

#### Respirometry

As described previously^23^, intestinal tissue was harvested at the end of the experiments, placed in 4 °C isolation buffer (200 mM mannitol, Carl Roth GmbH, Karlsruhe, Germany; 50 mM sucrose Carl Roth GmbH, Karlsruhe, Germany; 5 mM KH_2_PO_2_ Merck, Darmstadt, Germany; 5 mM 3-(*N*-morpholino)-propanesulfonic acid (3-MOPS) Carl Roth GmbH, Karlsruhe, Germany; 0.1% bovine serum albumin BSA, Sigma-Aldrich Corporation, St. Louis, USA; 1 mM ethylene glycol-bis-(beta-aminoethylether)-tetraacetic acid (EGTA), Carl Roth GmbH, Karlsruhe, Germany, pH 7.15) and cleaned gently from faeces and mucus. After incubation with 0.05% trypsin for 5 min to decompose interstitial components, samples were transferred into an isolation buffer containing protease inhibitors (Complete™ Protease Inhibitor Cocktail, Roche Life Science, Mannheim, Germany) and 2% BSA. Tissue blocks were homogenized (Potter-Elvehjem, Pro Scientific, Swedesboro, NJ, USA, 5 strokes, 2000 rpm) and protein concentration was determined^62^ with 1% bovine serum albumin (BSA, Sigma-Aldrich Corporation, St. Louis, MO, USA) as a standard. All procedures were performed on ice to inhibit intrinsic enzymatic activity.

Homogenates were suspended in respiration buffer (130 mM potassium chloride, 5 mM K_2_HPO_4_, 20 mM 3-(*N*-morpholino)-propanesulfonic acid (3-MOPS), 2.5 mM EGTA, 1 µM Na_4_P_2_O_7_ and 2% BSA, pH 7.15) and measurements were performed using a Clark-type electrode (model 782, Strathkelvin instruments, Glasgow, Scotland) at a protein concentration of 6 mg · ml^−1^. In detail, glutamate (Fluka Chemie GmbH Buchs, Switzerland, 2.5 mM) and malate (Serva Electrophoresis GmbH, Heidelberg, Germany, 2.5 mM) as substrates of mitochondrial complex I or succinate (Sigma-Aldrich Corporation, St. Louis, MO, USA, 5 mM) as a substrate of mitochondrial complex II were added to measure oxygen consumption for electron transfer within the respiratory chain (state 2). Before the addition of succinate, 0.5 µM rotenone (Sigma-Aldrich Corporation, St. Louis, USA) as a blocker for complex I was added. Further, the maximal mitochondrial respiration was measured by the addition of ADP (Sigma-Aldrich Corporation, St. Louis, MO, USA, 125 µM) (state 3). The coupling between the electron transfer system and the oxidative phosphorylation was determined by calculation of the respiratory control index (RCI) dividing state 3 through state 2. ADP/O-ratio (ADP added/0_2_ consumption in state 3) was determined to mirror the efficiency of oxidative phosphorylation. Respiration rates are expressed as nanomoles per minute per milligram protein (nmol·min^−1^·mg^−1^). Before the definitive measurement of mitochondrial respiration cytochrome c and oligomycin were added to check mitochondria for leakage of the outer and inner membrane.

### Malondialdehyde levels

Malondialdehyde (MDA) is a product of the degradation of polysaturated fatty acids by lipid peroxidation and serves as a biomarker of oxidative stress^63^. To determine ileal and colonic MDA concentration, frozen tissue samples were homogenized in 500 µl 1.5% KCl (Fluka Chemie GmbH, Buchs, Switzerland) and mixed with 1% phosphoric acid (Merck, Darmstadt, Germany) and 0.6% thiobarbituric acid (Merck, Darmstadt, Germany). Malondialdehyde and thiobarbituric acid form a product that can be measured spectrophotometrically at 535 nm and 520 nm. The homogenate is heated to 95 °C for 45 min and after cooling mixed with 2000 µl of butanol (Merck, Darmstadt, Germany). The obtained mixture was vortexed and centrifugated at 2900 rcf for 15 min to collect the supernatant for spectrophotometrical measurement at a wavelength of 535 nm and 520 nm^64^. MDA concentration was calculated using a standard curve and normalized to protein concentration. Values are reported as nanomole MDA per milligram protein^62^.

### Evaluation of intestinal tissue integrity

#### Plasmatic d-lactate concentration

Lactate, an acidic metabolite of carbohydrate degradation, exists in two stereoisomers. l-Lactate, a product of anaerobic cell respiration, is used diagnostically to detect severe shock states with a manifest failure in energy metabolism. Increased l-lactate plasma concentrations are strongly associated with poor outcome and increased mortality^65^. Its stereoisomer d-lactate is produced in minimal quantities by mammalian cells and occurs as a metabolic product of indigenous bacteria in the intestinal tract^66^. Therefore, increasing levels of plasmatic d-lactate concentration might mirror an intestinal bacterial overgrowth or the loss of intestinal barrier function due to local tissue injury^36^.

Plasmatic d-lactate concentration was measured with a commercially available colorimetric assay (d-lactate, colorimetric assay kit, MAK058-1KT, Sigma-Aldrich, Germany) to determine intestinal barrier function. Blood samples were taken at the end of the experiments, centrifugated (10 min, 7000 rcf) to obtain blood plasma and stored at − 80 °C. Finally, a colorimetric measurement at 450 nm wavelength is used to determine MTT-formazan concentration, which is stoichiometrically related to d-lactate concentration in the blood samples.

#### Histological examination

Ileal tissue blocks from about 1 cm length were harvested after the experiment for histological preparation. Tissue was cut into 8 µm slides to estimate total tissue damage. In a standard HE-staining, every slide was divided into five sections, which were scored separately by two blinded investigator. According to the Chiu-scoring system and its further development by Park et al. tissue integrity was described by a gradual loss of adherence between enterocytes and the subepithelial stroma^67,68^. All values were averaged to calculate the mean scoring value. Since histological slides are mainly used to describe tissue components and structuring, we used this method to determine structural integrity of the intestinal barrier. Since the Chiu-scoring system is only validated for ileum slides, histological evaluation was not performed in colon ascendens tissue samples.

### Statistics

An à priori power analysis (G*Power Version 3.1.9.2) was used to calculate the required group size. As improvement of µHbO_2_ was defined to be the primary endpoint in this study, a group size of n = 12 is needed to achieve a power > 0.8 for the detection of differences between the experimental groups with α < 0.05 and η^2^ of 0.5.

Data for micro- and macrocirculatory analysis were obtained during the last 5 min of each experimental period under steady-state conditions. Normal distribution was assessed by Q-Q-plots (IBM SPSS Statistics, International Business Machine Corp., United States). Further, a two-way analysis of variance for repeated measurements (ANOVA) combined with a Bonferroni post-hoc test (GraphPad Prism version 6.05 for Windows, GraphPad Software, La Jolla California United States) was used to test time-related differences and differences between the experimental groups. Microvascular flow index is a semiquantitative score, that would generally require non-parametric analysis. Like discussed elsewhere, we performed multiple measurements to mirror the physiological spectrum of microvascular blood flow with multiple intermediate flow stages^28^. Since d-lactate measurements were assessed once during the experiment, one-way analysis of variance and Sidak correction for multiple comparison between the experimental groups was used to analyze d-lactate values. All data are presented as absolute values of mean ± standard error of the mean for n = 12. Variables of mitochondrial respiration and MDA levels did not follow normal distribution. Therefore, data were analyzed using Kruskal–Wallis testing and Dunn’s multiple comparison. Values are reported as median ± interquantile range for n = 12.

## Data Availability

All data generated or analyzed during this study are included in this published manuscript or can be made available by the corresponding author upon request. In this case, please use the following address: Stefan.hof@med.uni-duesseldorf.de.
